# Genetics causal analysis of oral microbiome on type 2 diabetes in East Asian populations: a bidirectional two-sample Mendelian randomized study

**DOI:** 10.3389/fendo.2024.1452999

**Published:** 2024-08-23

**Authors:** Xinyi Lyu, Xueyuan Xu, Sihong Shen, Feng Qin

**Affiliations:** ^1^ West China Medical School, West China Hospital, Sichuan University, Chengdu, Sichuan, China; ^2^ Department of Endocrinology and Andrology Laboratory, West China Hospital, Sichuan University, Chengdu, Sichuan, China

**Keywords:** oral microbiome, type 2 diabetes, Mendelian randomization, genetic variation, causal inference

## Abstract

**Introduction:**

The dysbiosis of the oral microbiome is associated with the progression of various systemic diseases, including diabetes. However, the precise causal relationships remain elusive. This study aims to investigate the potential causal associations between oral microbiome and type 2 diabetes (T2D) using Mendelian randomization (MR) analyses.

**Methods:**

We conducted bidirectional two-sample MR analyses to investigate the impact of oral microbiome from saliva and the tongue T2D. This analysis was based on metagenome-genome-wide association studies (mgGWAS) summary statistics of the oral microbiome and a large meta-analysis of GWAS of T2D in East Asian populations. Additionally, we utilized the T2D GWAS summary statistics from the Biobank Japan (BBJ) project for replication. The MR methods employed included Wald ratio, inverse variance weighting (IVW), weighted median, MR-Egger, contamination mixture (ConMix), and robust adjusted profile score (RAPS).

**Results:**

Our MR analyses revealed genetic associations between specific bacterial species in the oral microbiome of saliva and tongue with T2D in East Asian populations. The MR results indicated that nine genera were shared by both saliva and tongue. Among these, the genera *Aggregatibacter*, *Pauljensenia*, and *Prevotella* were identified as risk factors for T2D. Conversely, the genera *Granulicatella* and *Haemophilus D* were found to be protective elements against T2D. However, different species within the genera *Catonella, Lachnoanaerobaculum, Streptococcus*, and *Saccharimonadaceae TM7x* exhibited multifaceted influences; some species were positively correlated with the risk of developing T2D, while others were negatively correlated.

**Discussion:**

This study utilized genetic variation tools to confirm the causal effect of specific oral microbiomes on T2D in East Asian populations. These findings provide valuable insights for the treatment and early screening of T2D, potentially informing more targeted and effective therapeutic strategies.

## 1 Introductions

Diabetes mellitus encompasses a range of metabolic disorders related to carbohydrate metabolism, marked by inadequate glucose utilization as an energy source and excessive glucose production due to abnormal gluconeogenesis and/or glycogenolysis, resulting in hyperglycemia (1). Approximately 537 million adults worldwide suffer from diabetes, with over 90% of these cases being type 2 diabetes (T2D), and this number is projected to increase to 783 million by 2045 (2). Recently, large population-based studies have shown that periodontal disease adversely affects glycemic control, diabetes complications, and the progression of T2D (3, 4). Treatment of oral diseases has been demonstrated to improve glycemic control and reduce HbA1c levels (5).

The oral microbiome is the second largest microbial community in the human body, following the gut microbiome. It comprises over 700 species of bacteria, fungi, viruses, and protozoa (6). These microorganisms colonize the teeth, prosthodontic surfaces, mucosal surfaces, and are abundant in saliva (7). The oral microbiome is integral not only to oral diseases such as caries and periodontitis but also to systemic health (8, 9). This influence is mediated through complex interactions with the host immune system, the gut microbiome, and various small molecule metabolites (10), and these interactions can impact systemic health by inhibiting pathogens, modulating the immune response, and affecting nutrient absorption and metabolism (11). Previous studies have indicated that dysbiosis of the oral microbiome is associated with the progression of various systemic diseases, including diabetes (12). For instance, certain periodontal pathogens, such as *Porphyromonas gingivalis* and *Aggregatibacter actinomycetemcomitans* have been directly linked to glycemic control and the risk of developing diabetes (13, 14).

However, compared to the extensive research on the gut microbiome, studies on the oral microbiome were relatively limited and often involved small sample sizes. Traditional observational studies may also suffer from inadequate control of confounding variables and the possibility of reverse causation. Therefore, we employed Mendelian randomization (MR) as an epidemiological tool (15). MR leverages naturally randomized genetic variants at conception as a form of natural experiment to uncover causal relationships between exposures and outcomes, thereby minimizing the potential for reverse causation and confounding biases (16, 17). In this study, we conducted bidirectional two-sample MR analyses to investigate the impact of oral microbiome from saliva and the tongue T2D using single nucleotide polymorphisms (SNPs) as instrumental variables.

## 2 Materials and methods

### 2.1 Study design and population

**Figure 1:**
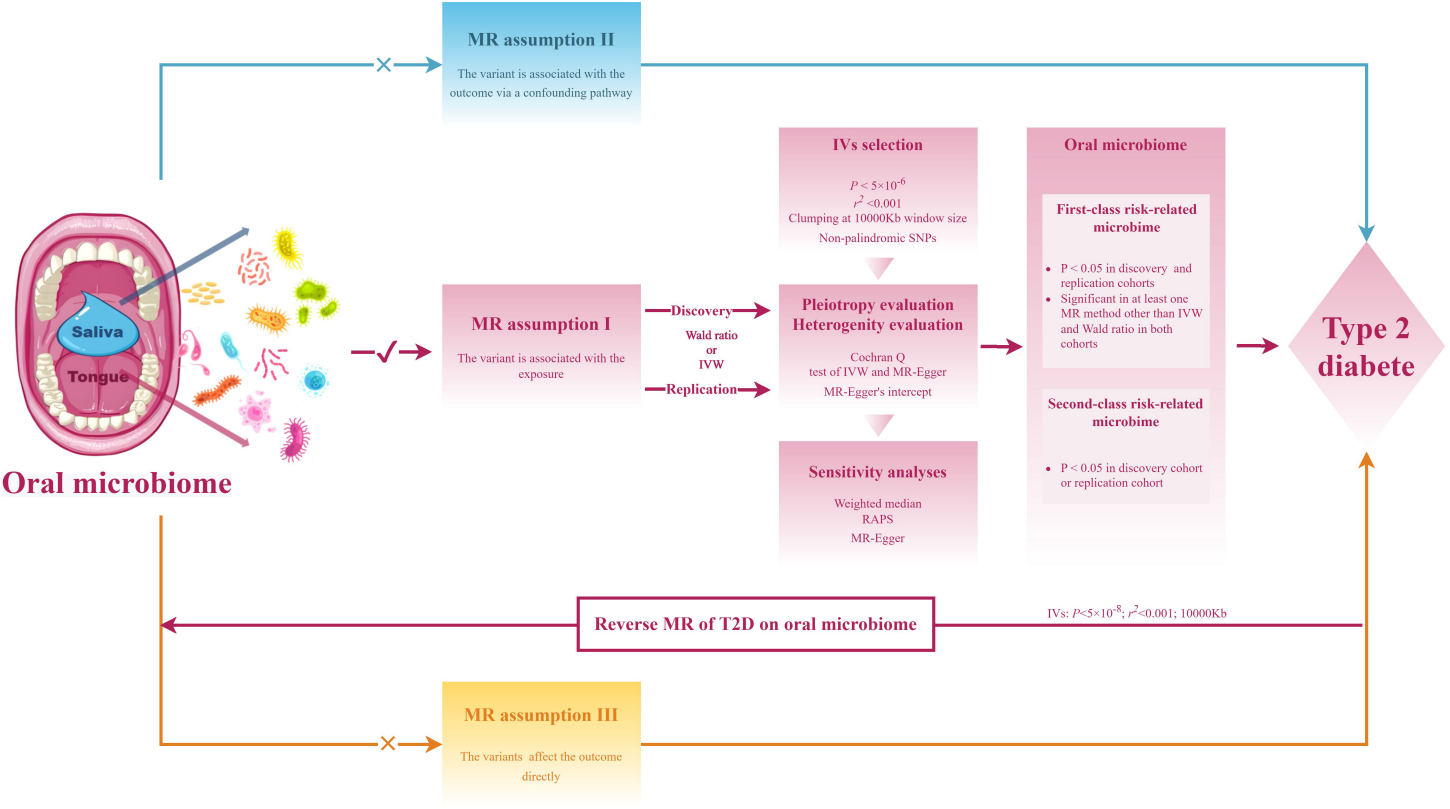
Flowchart of the study design.

The research workflow is presented in **Figure 1**. The summary statistics for the metagenome-genome-wide association studies (mgGWAS) of the oral microbiome in East Asian populations were obtained from the research by Liu et al. (18), This study comprised 2017 tongue samples and 1915 salivary samples, derived from a cohort of 2984 healthy Chinese individuals with provided high-depth whole genome sequencing data. In this study, the lowest taxonomic level is species. The composition of the microbiome used in the study was determined by aligning it against 56,213 metagenome-assembled genomes (MAGs), which were organized into 3,589 species-level clusters (SGBs). The criteria for sample inclusion were as follows: a) a variant calling rate exceeding 98%; b) an average sequencing depth greater than 20×; c) no population stratification as evidenced by principal component analysis (PCA); d) the exclusion of related individuals based on pairwise identity by descent calculations. Additionally, a stringent inclusion threshold was applied for variants, requiring a mean depth greater than 8×, Hardy-Weinberg equilibrium (HWE) P-value greater than 10^−5^, and a genotype calling rate exceeding 98%.

Summary statistics of T2D were obtained from meta-analyses of GWAS conducted by Spracklen et al (19), which encompassed 77418 T2D cases and 356122 controls (effective sample size, Neff=211793) of East Asian individuals from 23 GWAS, For each study involved in this meta-analysis, variants were excluded according to the following criteria: a) mismatched chromosomal positions or alleles compared to the reference panel; b) ambiguous alleles (AT/CG) with a minor allele frequency (MAF) greater than 40% in the reference panel; or c) discrepancies in allele frequencies exceeding 20% when compared to East Asian-specific allele frequencies. This study was designated as the discovery cohort. Furthermore, we utilized the T2D GWAS summary statistics from the Biobank Japan (BBJ) project as a replication cohort (20), comprising 45383 T2D cases and 132032 controls of East Asian individuals. There is no sample overlap between the exposure and the outcome. The details of these datasets are shown in **Supplementary Table 1**.

### 2.2 Instrumental variables selection

In MR analysis, we employed the PLINK clumping (21) function to identify a sufficient number of independent instrumental variables (IV), which reduces linkage disequilibrium (LD) among associated genetic variants, avoids multicollinearity due to LD, and mitigates biases caused by weak instruments. Initially, we selected instrumental variables based on a genome-wide significance threshold of *P <*5×10^-8^ to test our hypothesis. However, due to the limited number of instruments available at this threshold, we adopted a more lenient threshold to ensure the robustness of our analysis: p1 = 5×10^-6^, p2 = 1×10^-5^, kb=10000Kb, and r2 = 0.001, to identify top loci. SNPs with a minor allele frequency (MAF) < 0.01 are generally considered rare SNPs, which have a limited impact on traits. Hence, only SNPs with MAF ≥ 0.01 were retained. Additionally, we applied Steiger filtering (22) to the instrumental variables and excluded instruments with F-statistics (F= (beta/se) ^2^) < 10 to mitigate the impact of weak instrumental variables (23).

### 2.3 Bidirectional MR analysis

We utilized six MR analysis methods to investigate the impact of oral microbiome from saliva and tongue dorsum on T2D, including Wald ratio, inverse variance weighting (IVW) (17), weighted median (WM) (24), MR-Egger (25), Contamination mixture (ConMix) (26), robust adjusted profile score (RAPS) (27). For MR analyses with only one instrumental variable, we employed the Wald ratio as the primary analysis method. Additionally, as the RAPS method could produce consistent results in the presence of weak and pleiotropic SNPs, it was utilized for supplementary validation in MR analyses with only one instrumental variable. IVW was chosen as the primary analysis method for MR analyses with multiple instrumental variables, given its robustness. The ConMix method explicitly models multiple potential causal estimates and infers various causal mechanisms linked to the same risk factor, each impacting the outcome to different extents. MR-Egger regression offers estimates corrected for pleiotropy. The WM estimator, which calculates the median of the weighted estimates, provides a consistent effect even when up to half of the instrumental variables are pleiotropic. We applied FDR for multiple testing correction, with FDR<0.05 indicating significance and P < 0.05 suggestive significance.

We conducted various heterogeneity and pleiotropy analyses to evaluate the robustness of our results against potential violations of multiple MR assumptions. a) Heterogeneity was assessed through the Cochran Q test of IVW and MR-Egger methods; b) Horizontal pleiotropy was evaluated using MR-Egger’s intercept. The same approach was applied to reverse MR analyses to mitigate spurious results arising from reverse causation. Additionally, a stricter threshold was applied for instrumental variables with T2D as the exposure (p1 = 5×10^-8^, p2 = 1×10^-5^, kb=10000Kb, r2 = 0.001) to enhance result reliability.

All the analyses were conducted using R software 4.2.0. The IVW, MR–Egger, WM, MR-RAPS, and ConMix methods were performed using the “TwoSampleMR” package.

## 3 Results

### 3.1 Causal effects of oral microbiome in the saliva on the development of T2D

**Figure 2:**
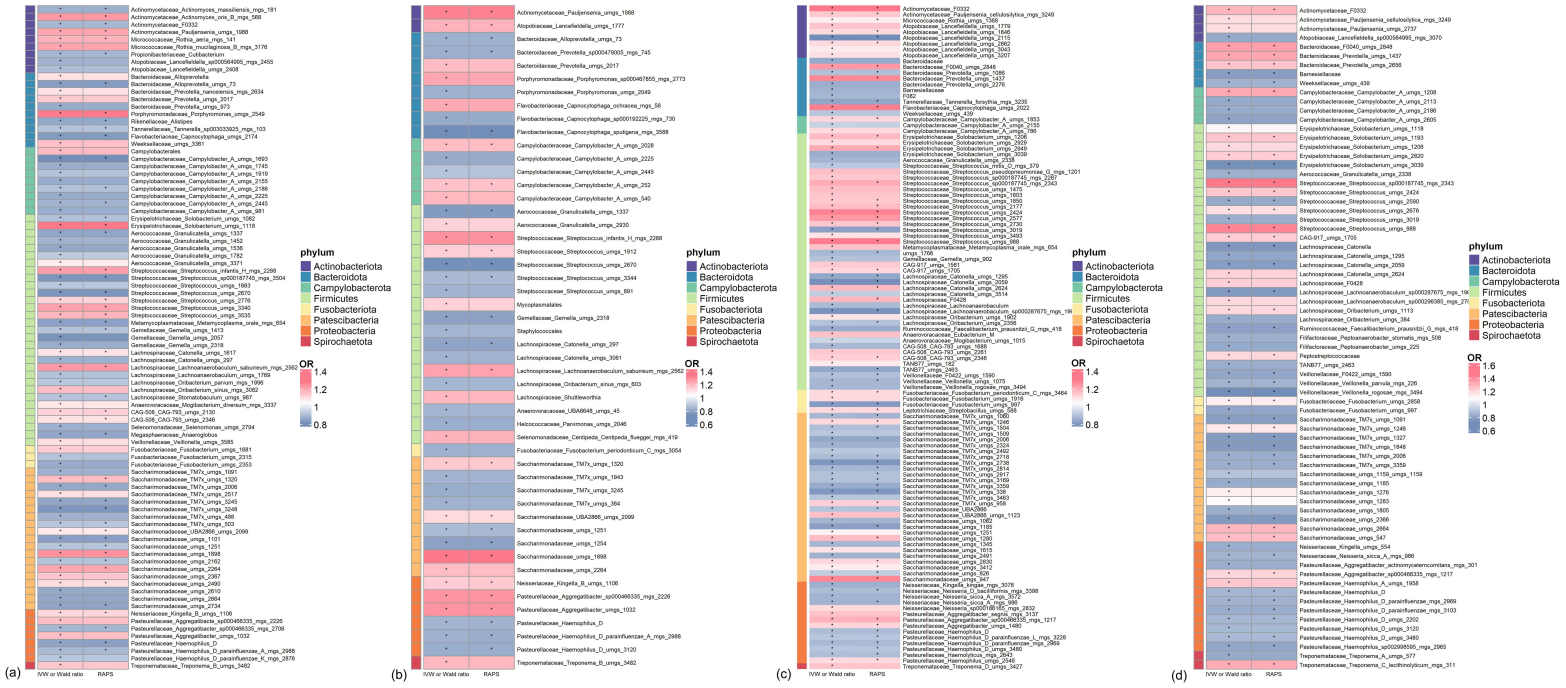
Significant mendelian randomization (MR) results of microbiome in saliva and tongue coating for type 2 diabetes (T2D) in discovery and replication Cohorts. **(A)** Oral microbiome in saliva showing significant MR results in the discovery cohort. **(B)** Oral microbiome in saliva showing significant MR results in the replication cohort. **(C)** Oral microbiome on the tongue showing significant MR results in the discovery cohort. **(D)** Oral microbiome on the tongue showing significant MR results in the replication cohort. * indicates MR P-value < 0.05.

All genetic instruments used in the MR analyses passed the Steiger test. Additionally, the F-statistics for these instruments were greater than 10, indicating strong instrument validity. (**Supplementary Tables 2**, **3**). In the discovery cohort of T2D, a total of 89 bacterial species in saliva (46 genera, 28 families, 20 orders, 11 classes, and 8 phyla) had statistically significant relationships (P < 0.05) with T2D under either the IVW or Wald ratio MR methods. Among these, 48 bacterial species were confirmed to be significant under the RAP method, with consistent effect directions (**Figure 2A**; **Supplementary Table 4**). In the replication cohort of T2D, a total of 50 bacterial species in saliva (29 genera, 19 families, 17 orders, 11 classes, and 8 phyla) had statistically significant relationships (P < 0.05) with T2D under either the IVW or Wald ratio MR analysis methods. Among these, 26 bacterial species were confirmed to be significant under the RAP MR analysis method, with consistent effect directions (**Figure 2B**; **Supplementary Table 5**).

In both the discovery and replication cohorts, 23 bacterial species were consistently significant under either the IVW or Wald ratio MR methods. Of these, 12 species were positively correlated with the risk of developing T2D (OR > 1), while 11 species were negatively correlated (OR < 1) (**Supplementary Table 6**). We further examined whether the instrumental variables corresponding to these 23 bacteria were associated with other confounding factors. We found that rs10421891, the instrumental variable for *Prevotella unclassified metagenome species* (uMGS) 2017, has been reported to exhibit genome-wide significance about heart failure and left ventricular systolic function phenotypes. No associations with other confounding factors were identified for the remaining instrumental variables (**Supplementary Table 7**).

**Figure 3:**
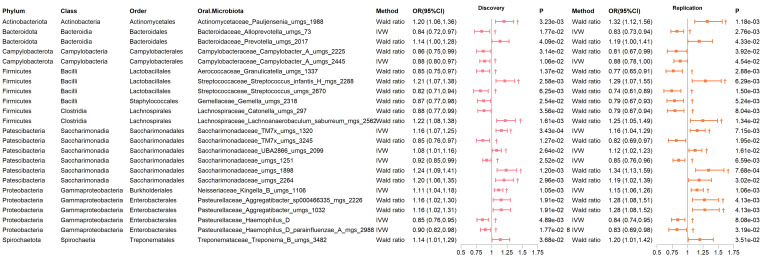
Significant MR results of oral microbiome in saliva for T2D in both discovery and replication Cohorts. † indicates a P-value < 0.05 in at least one MR method other than IVW or Wald ratio.

Notably, in the discovery and replication cohorts, several genera showed significant relationships with T2D risk in at least one MR analysis method other than IVW and Wald ratio. Except for the MR Egger analysis of *Haemophilus D*, which indicated some heterogeneity (Q_Egger_=4.239, P_heterogeneity_=3.95×10^-2^), the remaining sensitivity analyses of the MR showed no significant heterogeneity or pleiotropy (**Figure 3**; **Supplementary Table 6**). These included *Pauljensenia, Streptococcus, Lachnoanaerobaculum, Saccharimonadaceae TM7x, Saccharimonadaceae UBA2866, and Saccharimonadaceae uMGS 1898*, which were positively correlated with T2D risk, as well as *Alloprevotella, Granulicatella, Streptococcus, Saccharimonadaceae uMGS 1251, and Haemophilus D*, which were negatively correlated with T2D risk. Reverse MR analysis did not reveal any causal relationships (all P > 0.05, **Supplementary Table 8**).

### 3.2 Causal effects of oral microbiome in the tongue on the development of T2D

All genetic instruments employed for MR analyses successfully passed the Steiger test. Furthermore, the F-statistics of these genetic instruments exceeded 10, demonstrating robust instrument strength (**Supplementary Tables 9**, **10**). In the discovery cohort of T2D, we identified 114 bacterial species in saliva (55 genera, 30 families, 21 orders, 11 classes, and 8 phyla) that exhibited statistically significant associations (P < 0.05) with T2D when analyzed using either the IVW or Wald ratio MR methods. Of these, 66 bacterial species were further validated as significant under the RAP method, maintaining consistent effect directions (**Figure 2C**; **Supplementary Table 11**). Similarly, in the replication cohort of T2D, 73 bacterial species in saliva (spanning 40 genera, 21 families, 18 orders, 11 classes, and 8 phyla) showed statistically significant associations (P < 0.05) with T2D using IVW or Wald Ratio. Out of these, 44 bacterial species were confirmed as significant via the RAP method, with effect directions remaining consistent (**Figure 2D**; **Supplementary Table 12**).

**Figure 4:**
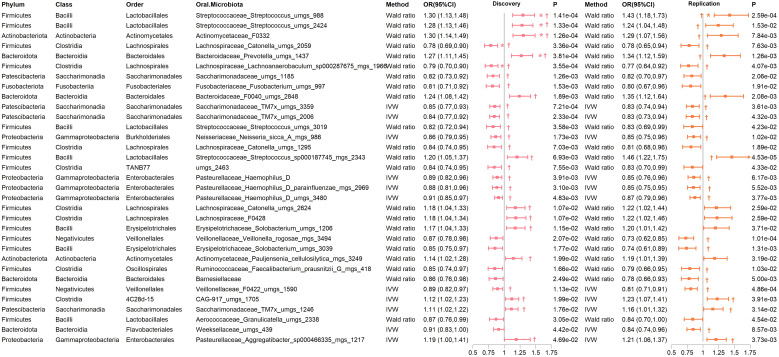
Significant MR results of oral microbiome on the tongue for T2D in both discovery and replication cohorts. † indicates a P-value < 0.05 in at least one MR method other than IVW or Wald ratio. * indicates that the FDR of the MR is less than 0.05.

In both the discovery and replication cohorts, 33 bacterial species were consistently significant under either the IVW or Wald ratio MR methods. Within this group, 13 species were positively associated with the risk of developing T2D (OR > 1), whereas 20 species were negatively associated (OR < 1) (**Supplementary Table 13**). To assess potential associations with other confounding factors, we analyzed the instrumental variables for these 33 bacteria. Our findings indicated that rs4566929, the instrumental variable representing *Streptococcus uMGS 2424*, has shown genome-wide significance for the body weight phenotype (**Supplementary Table 7**). For the other instrumental variables, no significant associations with additional confounding factors were detected. Notably, six species remained significant in the MR analysis after FDR multiple corrections (**Figure 4**; **Supplementary Table 13**). These include *Actinomycetaceae F0332 (genera), Streptococcus uMGS 988, Streptococcus uMGS 2424, and Prevotella uMGS 143*7, all of which are positively associated with T2D risk (OR >1), and *Catonella uMGS 2059 and Lachnoanaerobaculum sp000287675 MGS 1966*, both of which are negatively associated with T2D risk (OR < 1).

Importantly, several genera demonstrated significant associations with T2D risk in at least one MR method other than IVW and Wald ratio in both cohorts. These included *Actinomycetaceae F0332, Bacteroidaceae F0040, Prevotella, CAG-917 uMGS 1705, Aggregatibacter, Saccharimonadaceae TM7x, and Streptococcus*, which were positively correlated with T2D risk. Conversely, *Catonella, Lachnoanaerobaculum, Neisseria, Haemophilus D, Saccharimonadaceae TM7x, and Veillonellaceae F0422* were negatively correlated with T2D risk (**Figure 4**; **Supplementary Table 13**). Reverse MR analysis did not reveal any causal relationships (all P > 0.05). The sensitivity analysis of the MR indicated no significant heterogeneity or pleiotropy (**Supplementary Table 14**).

Notably, nine genera were shared by both the saliva and tongue, including *Aggregatibacter, Catonella, Granulicatella, Haemophilus D, Lachnoanaerobaculum, Pauljensenia, Prevotella, Streptococcus and Saccharimonadaceae TM7x* (**Supplementary Table 15**).

## 4 Discussion

Oral microorganisms are integral to the development and progression of oral diseases such as dental caries and periodontitis through mechanisms of pathogen inhibition and immune modulation (1, 2). Additionally, the oral microbiome contributes to overall systemic health by regulating immune responses, aiding nutrient absorption, and metabolism, and has been closely linked to systemic diseases such as T2D (28, 29). Despite this, the precise mechanisms and genetic causal relationships remain poorly understood. This study employs MR analysis to elucidate the genetic connections between the oral microbiome and T2D.

Aemaimanan et al. indicated that poor glycemic control is associated with increasing cell numbers of the red complex bacteria (*Porphyromonas gingivalis, Treponema denticola, and Tannerella forsythia*) within the subgingival biofilm (30). Li et al. also identified that periodontal pathogens, including *Porphyromonas gingivalis, Treponema denticola, and Fusobacterium nucleatum*, are significantly more abundant in T2D patients compared to normal controls. Furthermore, the *Firmicutes/Bacteroidetes (F/B)* ratio was higher in T2D patients than in healthy individuals (11), which aligns with the observed trend in newly diagnosed diabetes patients, where there is a decrease in the abundance of *Bacteroidetes* and an increase in the abundance of *Firmicutes* in the gut microbiome (31). Previous studies have found that an increase in *Firmicutes* or a higher *F/B* ratio is associated with obesity, a risk factor for T2D, as *Firmicutes* are more efficient than *Bacteroidetes* at extracting energy from food (32). Chen et al. found that the *F/B* ratio increased in patients with T2D, suggesting that this ratio may serve as a specific microbial biomarker in Chinese patients with T2D (33). Our findings also revealed that oral microbiomes genetically linked to T2D were predominantly *Firmicutes*.

Lu et al. observed that *Treponema, Prevotella oralis*, and *Catonella* were more abundant in the group with periodontitis and diabetes compared to the systemically healthy group (34). Additionally, *Prevotella* is more commonly found in the gut microbiome of T2D patients (35). We also confirmed that *Prevotella* in the oral microbiome is positively correlated with T2D. Previous studies have shown that *Prevotella* is associated with increased production of branched-chain amino acids (BCAAs). Elevated levels of BCAAs in the blood over the long term are linked to a higher risk of obesity and T2D (36). While *Treponema* in saliva showed a significant negative correlation. Regarding *Catonella*, certain species have shown a positive correlation with T2D, while more species have been found to have a negative correlation with the risk of developing T2D. *Aggregatibacter actinomycetemcomitans (A.a)* has been found to disrupt host mucosal defenses and was identified as one of the pathogenic bacteria involved in periodontitis (37). Castrillon et al. demonstrated that *A. a* detection was higher in patients with diabetes and periodontitis than in systemically healthy patients without periodontitis, with *A.a* being associated with periodontitis in diabetic patients (14). Our research confirmed a positive correlation between *Aggregatibacter* and T2D risk. *A.a* possesses some putative virulence factors, including leukotoxin that targets and destroys host immune cells. Previous reports have associated *A.a* with adverse events such as cerebral infarction in diabetic nephropathy patients undergoing hemodialysis (38). Furthermore, compared to control mice, mice infected with *A.a* exhibit impaired glucose tolerance and insulin resistance, along with alterations in the composition of their gut microbiota (39). *Pauljensenia*, a Gram-positive, strictly anaerobic, non-spore-forming bacterium from the family *Actinomycetaceae*. Previous studies have identified *Actinomycetaceae* as an oral biomarker for T2D (10). We further substantiated the positive correlation between *Actinomycetaceae* presence in both saliva and tongue and the risk of developing T2D. Its pathogenic role may be associated with glucose metabolism, participating in glycolysis for energy production, and accumulation of intracellular polysaccharides (40, 41), potentially increasing diabetes risk.

A study indicated a reduction in the presence of *Haemophilus* in the gut of patients diagnosed with T2D (31). The genus *Haemophilus* in the gut was identified as a defensive element against T2D (42). This finding is consistent with our study, which demonstrated a negative correlation between oral *Haemophilus D* and the incidence of T2D. Neri Rosario et al. employed machine learning techniques to identify *Granulicatella* and *Prevotella* as relevant genera in patients with prediabetes when compared to normoglycemic subjects (43). Previous studies have identified a negative correlation between the Plaque Index (PLI) and *TM7x* in patients with T2D and periodontitis (44). This suggests that *TM7x* may inhibit plaque formation or promote plaque clearance, potentially due to the defensive properties of certain *TM7x* species against T2D. *Streptococcus* has been identified as a marker bacterium in the oral and gut microbiome of patients with T2D (10). A study conducted on T2D patients in southern Thailand reported significantly higher total counts of salivary and plaque streptococci in diabetics compared to non-diabetics (45). *Streptococcus* is one of the earliest colonizers of the human body, particularly abundant in the oral cavity (46). It has been reported that pregnant individuals with pregestational diabetes with worse glycemic control were at an increased risk of group B streptococcus (GBS) colonization (46). In our study, various *Streptococcus* species exhibited mixed genetic causal effects on T2D, highlighting the complexity of the relationship between these bacteria and diabetes.

This study has several limitations. First, our MR analysis is concentrated on populations of East Asian ancestry, and additional validation is necessary to extend these findings to other ethnic groups. Second, factors beyond genetics, such as lifestyle, diet, and environmental influences, can also affect the oral microbiome (47, 48). The instrumental variables may explain only a small fraction of the observed variability, highlighting the need for further multidimensional research to fully comprehend the complex dynamics of the oral microbiome. Third, to ensure a sufficient number of SNPs as instrumental variables for the oral microbiome, we adopted a relatively lenient clumping threshold. Although various MR methods were used for sensitivity analysis and multiple corrections were applied to the results, the potential for some false positives cannot be entirely excluded.

## Data Availability

The original contributions presented in the study are included in the article/**Supplementary Material**. Further inquiries can be directed to the corresponding author.
